# The status of supergenes in the 21st century: recombination suppression in Batesian mimicry and sex chromosomes and other complex adaptations

**DOI:** 10.1111/eva.12291

**Published:** 2015-08-06

**Authors:** Deborah Charlesworth

**Affiliations:** ^1^ Institute of Evolutionary BiologyUniversity of EdinburghEdinburghUK

**Keywords:** Batesian mimicry, distyly, recombination suppression, sex chromosomes, sex‐limited expression

## Abstract

I review theoretical models for the evolution of supergenes in the cases of Batesian mimicry in butterflies, distylous plants and sex chromosomes. For each of these systems, I outline the genetic evidence that led to the proposal that they involve multiple genes that interact during ‘complex adaptations’, and at which the mutations involved are not unconditionally advantageous, but show advantages that trade‐off against some disadvantages. I describe recent molecular genetic studies of these systems and questions they raise about the evolution of suppressed recombination. Nonrecombining regions of sex chromosomes have long been known, but it is not yet fully understood why recombination suppression repeatedly evolved in systems in distantly related taxa, but does not always evolve. Recent studies of distylous plants are tending to support the existence of recombination‐suppressed genome regions, which may include modest numbers of genes and resemble recently evolved sex‐linked regions. For Batesian mimicry, however, molecular genetic work in two butterfly species suggests a new supergene scenario, with a single gene mutating to produce initial adaptive phenotypes, perhaps followed by modifiers specifically refining and perfecting the new phenotype.

## Introduction

I first became interested in the evolution of recombination rates when I was unemployed, after a postdoc working in human genetics, and started working as an unpaid research assistant to my husband, Brian Charlesworth. Brian asked me to help him with a computer program to check the accuracy of some equations he had derived concerning selection for chromosomal inversions through their effects on preventing genetic recombination. We showed that selection pressure for newly arisen inversions depends on the existence of a stable equilibrium with linkage disequilibrium (LD; Charlesworth and Charlesworth 1973). Much to my surprise, I have worked ever since on situations where suppressed recombination has evolved, or is predicted to do so, although the topic has also led me to study other evolutionary questions as well. Equally surprising is that, although there has been great progress on these situations, many important questions still remain to be answered.

More sophisticated treatments have since extended the modelling from studying invasion of populations by inversions to studying the behaviour of modifier mutations and alleles that control recombination rates. These theoretical models predict that lower recombination rates are often favoured by natural selection, and that epistatic interactions can lead to selection for decreased recombination in many natural situations (Otto and Lenormand 2002). The challenge has thus been to explain the maintenance of recombination, given that genetic variation exists at loci that affect recombination rates, often called ‘recombination modifiers’, and crossover frequencies differ between strains of maize and *Arabidopsis thaliana* (Sanchez‐Moran et al. 2002; Bauer et al. 2013). Recombination rates can therefore certainly evolve, and artificial selection experiments have produced changes in rates in *Drosophila melanogaster*, even in the absence of inversion differences, implying that recombination modifiers exist (Charlesworth et al. 1985; Brooks 1988; Korol and Iliadi 1994). Moreover, rates differ between closely related species and correlate with ecological factors, including long development times in mammals (Burt and Bell 1987), and high self‐fertilizing rates in plants (Roze and Lenormand 2005). Hotspots differ between humans and chimpanzees (Winckler et al. 2005; Auton et al. 2012), and genetic map distances differ between *Drosophila* species (Gubenko and Evgenev 1984; True et al. 1996) and mouse species (Dumont et al. 2010).

A large body of theory has been developed about the evolutionary forces that maintain recombination in most organisms and in most of their genomes (recently reviewed in Agrawal 2006; Charlesworth et al. 2009). Suppressed recombination suggests that unusual evolutionary forces have acted. In general terms, the evolution of suppressed recombination requires situations in which recombinant genotypes suffer some disadvantage compared with nonrecombinants. I will review three such real‐life situations that I have studied, all of which have been thought likely to involve the ‘supergene’ hypothesis, which is explained in Box 2. The cases examined in detail are sex chromosomes, Batesian mimicry and the control of two different flower morphs in distylous plants.

Box 1 **Personal reflections**

I have been interested in biology since childhood, but never imagined working as a scientist – in fact I had no idea that research jobs existed, and thought that laboratories were in hospitals and for teaching, and I imagined that I would like to be a technician in such a laboratory, like the ones at my school, where I enjoyed helping the technicians things get ready for classes. My parents were generally encouraging about my interest in science although their main interests were quite different. The idea of going to university came from my wonderful physics and maths teachers. Just before I started at Cambridge (UK), my mother warned me not to let any male friends know that I liked physics and maths! Luckily for me, I changed to biology right at the start, and luckily the course included at least some of the things that I loved (although not much about my chief interests, genetics and biophysics), and I met a student who had similar interests (we are still married). At Cambridge, the proportion of female students in science courses was low (even lower than the roughly 10% representation of students from the womens’ colleges overall, which limited the total numbers of women students until the other colleges started to admit women too), and the womens’ colleges were among the less rich colleges, so that we had fewer tutorials than most male students. However, by then, these moderate disadvantages, and the odd faculty member who tried to ignore female students, merely seemed ridiculous, and I we expected them to disappear shortly (which they did).

I shall always be grateful to the pioneers of higher education for women who founded college (Newnham), whose pictures were in the corridors, as my generation could study as full members of the university, with none of the battles that these inspiring women had been forced to fight (a favourite book is Ray Strachey's ‘The Cause’ – I love to give copies of it, and, sadly, cheap copies are often available as they get thrown out of university libraries). Awareness that some sorts of thinking were considered more suitable for men probably encouraged my interest in parts of biology that included some mathematical content (annoyingly, Cambridge biology students were not allowed to take a maths course, or even a statistics course). I do not feel that I have encountered serious prejudice in my subsequent career, since a couple of astonishing episodes of explicit prejudice in about 1970, and I think I have been lucky, because I believe that genetics and evolutionary biology have been particularly open towards women scientists, compared with other areas of science. However, subtle disadvantages certainly still exist, and probably have cumulative effects that can amount to serious obstacles, and I think it is it important to continue work to help young people of both sexes understand that opportunities should be open to both sexes. The lesson of history is that gains cannot be taken for granted, and I worry about the future for young scientists. The modern, highly competitive, often aggressive, atmosphere in science inevitably makes it difficult for scientists to slow down while they have young children, or if they have other family affairs to manage. This affects both men and women scientists, but it is highly likely to affect women most. I believe that working fewer hours can actually benefit research, so long as the actual working hours allow time for thinking – the ideas then sometimes mature in the ‘nonworking’ hours. Hours not actually at the desk or bench also allow time to think, and to plan ones’ day's work (I like to do this while walking the dog). I also find that ‘stepping back’ from the actual work helps provides some perspective about where it fits in with biology in general. We should therefore give more recognition to the need for young scientists to have thinking time and allow them to develop their creativity – this is currently in danger of being crushed, not only because of the demands on individuals of their own teaching and administrative tasks (teaching actually helps think and helps see one's research in perspective), but also because of the pressures to ‘deliver’ results, so that pressures are passed on to postdocs and students, impeding their intellectual development.

Box 2 **The evolution of closely linked gene clusters and the definition of a supergene**

A supergene is a system of closely linked loci controlling a polymorphic phenotype, such that a nonrecombining genome region is structured into two or more distinct haplotypes, each carrying a set of alleles that control multiple aspects of one of the phenotypes (see the figure below). The initial polymorphism is thought to evolve because an adaptive mutation arises, but fixation in the populations is prevented by some accompanying disadvantage. A second mutation subsequently arises that interacts with the first mutant allele, producing a phenotype with higher fitness, but reducing fitness when combined with the ancestral allele of the first gene.

A central concept is that the mutations are not unconditionally advantageous, but that their fitness effects depend on the genotype at the other locus or loci affecting the individuals. Assuming that the phenotypic effect of the second mutation is expressed in all individuals, irrespective of the allele at the first gene (rather than being expressed specifically only in the genotype that benefits, and not in the one that suffers the fitness cost, a possibility that is discussed in the main text), recombinant genotypes with some combinations of alleles are disadvantageous, as illustrated in the figure. This has two consequences. First, the second mutation may fail to spread in the population unless it occurs in a gene closely enough linked to the first gene. Second, if it does increase, selection favours less recombination, and suppressed recombination may evolve (as in part C of the figure), structuring the genome region into two haplotypes. Before suppressed recombination evolves, alleles at the loci in the system will show associations (linkage disequilibrium), with the disfavoured combinations becoming rarer after selection each generation. If complete linkage evolves, the disfavoured combinations are no longer generated.

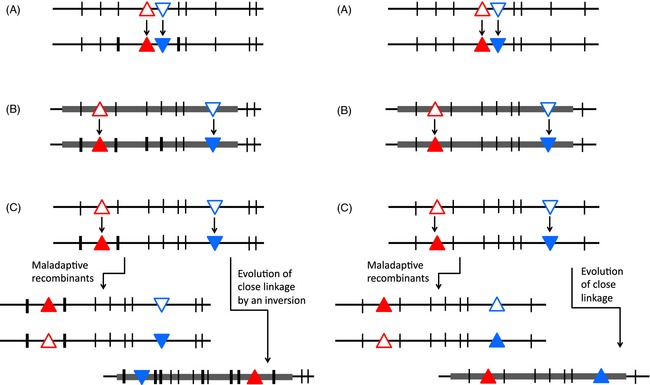

**Figure B2.** The evolution of closely linked gene clusters. An adaptation is shown involving changes at two genes (blue and red triangles changing from open to filled) in a genome region symbolized by a horizontal line. Other genes and neutral variants in the region are indicated by vertical lines. (A) A phenotypic change involving two closely linked interacting genes; close linkage does not require the evolution of suppressed recombination. Thick vertical lines indicate neutral variants that initially happened to be associated with the new adaptive haplotype. (B) Change involving two genes separated by many other genes in a nonrecombining genome region; again, close linkage does not require the evolution of suppressed recombination. (C) Change involving two genes separated by many other genes in a recombining genome region; close linkage requires the evolution of suppressed recombination after the adaptive changes (in the example shown, an inversion is illustrated). In this case, there are more thick vertical lines to indicate that new neutral variants have arisen in one haplotype, adding to the initial differences from the ancestral haplotype.

It is important to understand that not all such situations necessarily evolved suppressed recombination; sometimes, the loci may already have been linked before the polymorphism arose (cases A and B in the figure). One might well consider such cases supergenes, as they involve complexes of linked alleles (Schwander et al. 2014). Here, however, I do not discuss in detail cases where the evolution of suppressed recombination is not involved, which often involve adjacent loci (as in the case of plant homomorphic self‐incompatibility outlined in my article). Instead, I focus on cases like the ‘classical supergene’ situation C in the figure; these are thought to involve a change in recombination, linking many genes into nonrecombining haplotype blocks, or ‘complexes’, very few of which are involved in maintaining the polymorphisms whose interactions selected for suppressed recombination.

In the case of sex chromosomes, there is good evidence that suppressed recombination has indeed evolved, and the challenge is to understand why it did so, and particularly why this has evolved repeatedly in the sex chromosomes of animal and plant lineages. In the case of Batesian mimicry, I developed a theoretical model that predicts that recombination suppression should evolve, based on developmental arguments that suggested the involvement of multiple genes. However, as outlined below, recent results suggest that no extensive nonrecombining region exists at the mimicry locus, and the developmental assumptions underlying the prediction that a supergene should evolve have been questioned. In the case of distyly, it is not yet certain whether the genome region controlling the two different flower morphs in distylous plants is nonrecombining region.

Table 1 summarizes some of the situations where selection favours reduced recombination rates between the genes involved, to highlight the similarities between different cases. After outlining the three systems on which I have myself worked, I briefly discuss other systems where nonrecombining genome regions appear to have evolved or might be predicted to evolve.

**Table 1:** Three biological situations where the evolution of supergenes has been proposed

Situation	Initial state	Genes involved		Disadvantages to recombinants
		First mutation	Second mutation	
Batesian mimicry	Nonmimetic	Nonmimetic → mimetic	Modifier of mimicry pattern (mimetic→ improved mimetic)	Nonmimetic, but with the modifier of mimicry making individuals more conspicuous to predators
Sex‐determination	Cosexual (hermaphroditic or monoecious)	Male‐sterility (cosexual → female)	Female suppressor (cosexual → male)	Neuter (male sterile with suppressor of femaleness)
Distyly	Nondistylous	Changed stigma position	Changed anther position	Stigma and anther positions that encourage within flower self‐pollination

## Batesian mimicry

Batesian mimics are palatable and undefended species that have evolved predation avoidance through resembling unpalatable or defended species called ‘model species’ (Clarke and Sheppard 1960a,b). Mimicry may be fixed in Batesian mimetic species, but is often polymorphic, with both mimics and nonmimetic individuals present within populations. In either polymorphic or nonpolymorphic situations, there may also be sexual dimorphism: often males remain nonmimetic, and only females are mimetic (Kunte 2009). The mimetic forms within populations may also differ regionally, depending on the presence and abundance of model species. This polymorphism allows the genetics of mimicry to be studied.

Another important advantage of butterflies for studying Batesian mimicry is that the ancestral state of the characters can sometimes be inferred. In many species, the male colour and wing pattern clearly resemble closely related species (Kunte 2009). When females are polymorphic for the nonmimetic form, this shows that the differences are not simply sex differences in phenotype, but involve ancestral and derived (mimetic) states. The wing patterns of mimics differ from those of the nonmimetic morph, sometimes in several details that provide a very close resemblance to the model species. Multiple characters differ from the nonmimetic state, including colours of wing regions, and sometimes colour of parts of the body. In the swallowtail species *Papilio memnon*, the presence or absence of tails on the hindwings is controlled by variation at the same locus (Clarke and Sheppard 1972), and in one race of *Papilio polytes,* the tailed mimetic versus tailless nonmimetic difference again behaves as an allele at the mimicry locus (Clarke and Sheppard 1977). These differences seem very unlikely to be due to mutations in a single gene, and in *Papilio dardanus* an unlinked locus indeed controls such differences (in this case, nonmimics are tailed and mimics tailless in most populations, see Clarke and Sheppard 1960a,b). It was therefore proposed that such mimicry is a complex phenotype that evolved in several steps, involving mutations in multiple genes. Behavioural differences also exist between the sexes of most Batesian mimetic species, with males flying in more open habitats than females (Wallace 1865), but there is currently no reliable evidence supporting statements that the mimetic morphs differ in behaviour (K. Kunte, personal communication)

However, when the genetics of mimicry was studied in several Batesian mimetic butterflies in the genus *Papilio*, a single locus appeared to be responsible. It was therefore proposed that close linkage has evolved between different genes, resulting in a supergene – a polymorphic closely linked genome region with genes controlling different mimetic pattern and colour elements (Clarke and Sheppard 1960a,b), as in part C of the figure in Box 2. The region was envisaged to be organized into distinct haplotypes, one with the nonmimicry alleles at each of the loci and others with sets of alleles controlling the different mimetic morphs. If recombination is rare, occasional recombinants might be seen, and indeed butterflies with phenotypes that could be recombinant have been observed (Clarke and Sheppard 1972, 1977).

It is appropriate to discuss mimicry before the other cases of supergenes, or possible supergenes, because mimicry illustrates the ‘classical supergene’ hypothesis particularly clearly. Both the selective forces leading to the polymorphic situations envisaged (see Box 2), and why they select for reduced recombination, are easily understood in this case. To model the evolution of mimicry, I assumed that multiple mutations with suitable phenotypes are unlikely to arise simultaneously; instead, a first mutation produced an initial mimic, followed by one or more further genetic changes involving mutations improving the initial mimicry (see Table 1). For the evolution of close linkage between different genes, it is crucial that polymorphic situations are established at both loci, to produce a selection pressure for lower recombination (Box 2). For the first mutation, it is likely that mimicry involves costs that often lead to polymorphism. This is because mimetic butterflies (and their models) tend to be more conspicuous in their native habitats than their nonmimetic conspecifics. The spread of the first mutation depends on how well its carriers resemble the model species, how great a ‘cost’ is paid, in terms of being more conspicuous and also on the distastefulness of the model species (Charlesworth and Charlesworth 1975a). The system also depends on the abundance of the model species, and the assumptions just outlined create frequency dependence (mimics gain most when they are rare, because predators encounter models more often than mimics, and learn to avoid the mimetic pattern), but also a number dependence (if the model species is abundant, predators will readily learn to avoid the mimetic pattern). A rare allele advantage that diminishes with increased frequency may therefore lead to a polymorphism, rather than the mimicry allele becoming fixed in the population.

If mimicry alleles become established in a species, either as polymorphisms or fixed differences from the ancestral state, further mutations improving resemblance to the model will often be favoured, but again such modifier alleles will generally increase conspicuousness to the predators, and both mimicry mutations can establish polymorphisms. Under the assumptions just outlined, recombinant genotypes expressing the modifier (second) mutation together with the nonmimetic ancestral genotype at the first locus will be conspicuous, but not mimetic.

**Figure 1:**
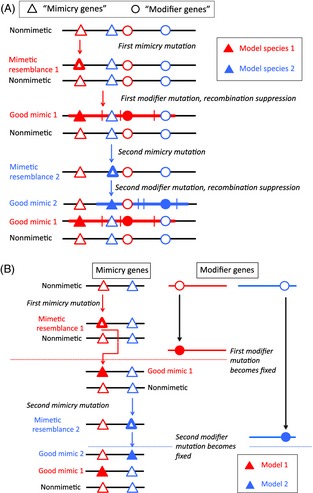
Two possible scenarios for the evolution of Batesian mimicry involving more than one gene. Alleles at mimicry genes are shown as triangles, and alleles of genes modifying mimicry as circles. (A) The classical (or original) supergene hypothesis. An initial mutation yields a mimetic resemblance (red open triangle) that confers enough protection against predation when rare, despite increasing conspicuousness to the predator, that it establishes a polymorphism in a population. A second mutation (red circle changed from open → closed) improves the mimetic resemblance (indicated by the red triangle becoming filled), at the cost of a further increase in conspicuousness; if it is closely enough linked to the first mimicry locus, it may spread and may establish a two‐gene polymorphism that selects for closer linkage, with haplotypes including both loci distinguishing the mimetic morph from the ancestral phenotype. Other mimetic morphs might evolve similarly (blue open and filled triangles and circles). If suppressed recombination evolves, the genome region will acquire variants specific to the different haplotypes (indicated by short vertical lines, and by the wide red or blue horizontal lines distinguishing the mimetic haplotypes from the ancestral one, shown as black). (B) The morph‐specific modifier alternative hypothesis with mimicry controlled by mutations in a single gene that determines some important aspects of wing pattern development. When a modifier is expressed in a mimetic morph, it becomes a good mimic, rather than having the poor mimetic resemblance initially caused by the mutation in the mimicry gene.

The population genetics of situations involving such interacting genes, where recombinants suffer reduced fitness, predicts that the invasion of the population by the second mutation will be affected by the recombination frequency between the two loci as well as its advantage in the presence of the first one (Charlesworth and Charlesworth 1975b). If the first mutation is polymorphic, a second one in a gene unlinked to the first locus can invade only if the benefits of better mimicry are large enough to outweigh the greater conspicuousness. Such mutations can spread to fixation, which also results in fixation of the first mimicry mutation, so that the improved mimetic form becomes fixed in the species (or just in females, if expression of the mimicry is sex‐limited). If, however, the second mutation is not so advantageous, it may increase in frequency and establish a polymorphism alongside that at the first locus, and, because one recombinant type is strongly disadvantageous, there is a selective pressure for closer linkage. This model can thus explain the evolution of a linked cluster of genes. For simplicity, I have described a succession of only two mutations contributing to mimicry, as in the figure in Box 2, but further changes of the same general type may well occur (see Fig. 1).

With modern molecular approaches, it is now becoming possible to identify the mimicry ‘locus’ and test the supergene theory that this genetic locus is, in reality, a cluster of closely linked genes. Indeed, in populations segregating for several different mimetic forms, several different mutations must have been involved. Recently, the mimicry loci have been identified in two swallowtail butterfly species with polymorphic Batesian mimicry, and the results do not appear to support the multigene supergene scenario in Box 2.

Two studies used *P. polytes*. In this species, the difference between the nonmimetic wing pattern (form *cyrus*) and one of the three mimetic patterns (confusingly named form *polytes*) was mapped to a genome region with only five genes that did not recombine in the family studied (Kunte et al. 2014; Nishikawa et al. 2015). If different genes with suitable mutational effects to produce mimicry have led to the evolution of suppressed recombination between them, one would expect other intervening genes, with no involvement in mimicry (possibly many genes as in part C of the figure in Box 2), to have become included within the nonrecombining region.

One of the genes linked to the mimicry locus, doublesex (*dsx*), is an interesting candidate for control of the mimetic patterns (Kunte et al. 2014). Indeed sequence variants identifying different *dsx* alleles are associated with mimicry locus alleles in wild‐caught butterflies, whereas the alleles determining mimetic versus nonmimetic phenotypes were not differentiated when variants in physically close (<800 kb) and closely linked loci on either side of *dsx* were examined (Nishikawa et al. 2015). Transgenic experiments also support the involvement of *dsx* in controlling the mimetic pattern studied (Nishikawa et al. 2015).

The discovery of the region allows a test of whether a nonrecombining region exists in the *P. polytes* mimicry region. It is difficult to test whether a genome region has evolved suppressed recombination, because the ancestral recombination rate is unknown, and recombination rates can differ between genotypes, as explained above. It is also difficult to test for LD in the genome region, because this requires information about the phase of variants (which variants are in which alleles of the sequences carried by individuals). Phase can be ascertained by laborious studies of parents and the alleles at different loci that are co‐inherited by different offspring (we shall see in the next section that things are simpler for sex‐linked genes). However, even without information about the phase of different variants, the supergene hypothesis is testable once the region near a candidate gene has been assembled. The basis for a test is that, as a consequence of selection against recombinants, other genes that are located in the same genome region, but not involved in the development of these patterns, may also be held in LD with alleles of the mimicry genes. In other words, haplotypes carrying different alleles at any one of the mimicry genes will tend to be associated with different alleles at all genes in the region (see the figure in Box 2). Therefore, the classical supergene hypothesis predicts high sequence diversity in the *P. polytes dsx* region, including flanking loci, not *dsx* alone. It is therefore of great interest to find out how far the apparent peak of polymorphism at *dsx* extends. Sequence diversity was found to be very high at *dsx*, significantly higher than in several nearby genes by HKA tests (Hudson et al. 1987), consistent with long‐term balancing selection acting on the *dsx* locus, as might be anticipated for a mimicry polymorphism (Kunte et al. 2014; Nishikawa et al. 2015). The alternative that the *polytes* allele has recently increased in frequency, causing a selective sweep in the region, is therefore unlikely, as this would predict low diversity in a set of such alleles sampled from natural populations.

The assembly of the wider *dsx* genome region is not yet complete for *P. polytes*, but the results obtained so far suggest that sequence diversity is very high only at *dsx*, implying that no nonrecombining region exists outside the *dsx* gene itself. Moreover, sequences of *cyrus* and *polytes* morphs (from individuals homozygous for the two different mimicry alleles) exhibited many fixed differences, particularly in exon 1 of *dsx*, whereas flanking loci did not (Kunte et al. 2014; Nishikawa et al. 2015).

There also appears to be no extended nonrecombining region around the mimicry locus in *P. dardanus* (Timmermans et al. 2014), although eight genes showed complete co‐segregation with mimicry alleles in the crosses studied, several involved in wing patterns and/or colours, including *engrailed* and *invected*. The conclusion that this is the mimicry region is strongly supported by the detection of associations between variants in these genes and five different mimicry alleles carried by individuals sampled from natural populations (particularly clearly at four loci). One morph appears to have a duplication of part of the region, and this could be contributing to the suppressed recombination, as no inversion has yet been found in this species; these genes, like *dsx*, are candidates for control of developmental processes.

The results just outlined suggest that variants within a single gene may control distinct wing pattern and colour elements in the Batesian mimics *P. dardanus* and *P. polytes*, and even perhaps the presence or absence of hindwing tails in *P. polytes*. This appears to contradict the developmental assumptions underlying the supergene prediction. However, it is important to understand that inheritance as a single locus does not mean that only one gene was involved in the evolution of mimicry. Fisher (1930) stressed that, although ‘a Mendelian factor …. decides between two (or more) alternatives, …. these alternatives may each be modified in the course of evolutionary development, so that the morphological contrast determined by the factor at a late stage may be quite unlike that which it determined at its first appearance’. In the context of mimicry, this idea proposes that an initial mimicry allele arose, followed by changes in the genetic background (including modifier mutations at loci unlinked to the initial mimicry gene) that improved the mimetic resemblance, without affecting the nonmimetic phenotype, so that improving the mimicry, does not impose any cost on the ancestral phenotype (Fig. 1B); there is then no selective force preventing fixation of the modifier, and no two‐gene polymorphism that selects for close linkage. As shown in the figure, similar changes at the same locus, followed by changes at loci elsewhere in the genome that specifically improve its resemblance to a local model species, could allow the evolution of further mimetic morphs.

This interpretation also invokes a succession of mutations, just as in the original supergene hypothesis outlined in Box 2 and illustrated in Fig. 1A for Batesian mimicry. It therefore does not represent a major change in how biologists believe complex phenotypes evolve. The difference lies in whether the genes that improve an initially imperfect phenotype express their effects in both initial types. In the classical (or original) supergene model, close linkage is a condition for the modifier alleles to spread in the population because the modifiers improving the mimicry are nonspecific, and closer linkage may subsequently evolve (see Box 2). In the new alternative model in Fig. 1B, recombination suppression has not evolved, but is a consequence of the variants involved in the adaptive changes being closely linked, simply because they are in the same gene (as in part A of Box 2), and specific modifiers, with effects restricted to some genotypes, may be involved. The single‐locus inheritance misleadingly suggests that the adaptation involved just one gene, whereas in fact, a complex adaptation, involving modifier mutations, occurred, but the modifier alleles have become fixed in the population. The distinction between specific and nonspecific modifiers will appear again in sex chromosome evolution.

Interestingly, however, there is also evidence suggesting evolution of suppressed recombination in mimicry loci of both the two Batesian mimics so far studied, which suggests that interactions may have occurred between successive mutations within a single locus (or in only a few very closely linked loci) and have led to the evolution of suppressed recombination. In *P. polytes*, inversions differentiate the two types of *dsx* sequences studied in Kunte et al. (2014), and a comparison with a close outgroup species, *Papilio xuthus*, indicated that a roughly 130 kb inversion occurred in the ancestry of the mimicry (*H*) allele, preventing recombination with the *dsx* allele carried in the nonmimetic (*h*) haplotype (Nishikawa et al. 2015). Three *P. polytes* transcripts were identified that had higher expression in the wings of mimetic than nonmimetic females and that are encoded by genes near the left breakpoint of the inversion in the assembly of the region. Intriguingly, the inversion changes the 5′ UTR and transcriptional start site for one of them, the transcriptional regulator, UXT, suggesting that the inversion may affect the regulation of neighbouring genes outside the inverted region (Nishikawa et al. 2015).

As explained above, there is also evidence for recombination suppression in *P. dardanus*, across a region including about eight genes, still a very small number. In another mimetic butterfly, *Heliconius numata*, chromosomal inversions were found at the mimicry locus, suppressing recombination over a 400 kb interval, and including at least 18 genes (Joron et al. 2011). However, this is believed to be a ‘Müllerian polymorphism’ (Joron et al. 2011), although it may also have elements of Batesian mimicry that could be contributing to maintaining the observed wing pattern polymorphism (Charlesworth and Charlesworth 2010); if so, this could represent a classical supergene, affecting somewhat larger, but still small, genome region.

## Sex chromosomes as supergenes

Unlike Batesian mimicry, the current view of the evolution of sex chromosomes closely fits the supergene scenario in the part C of the figure in Box 2, with large genome regions often having evolved suppressed recombination. The evolution of two sexes, and the differences between them, has recently been reviewed (Beukeboom and Perrin 2014) and is clearly a complex adaptation. When gender is genetically controlled, suppressed recombination has repeatedly evolved between the chromosome pairs that carry the sex‐determining genes. The X and Y chromosomes of mammals are familiar examples. Similar XY chromosome pairs are known in many other vertebrate taxa, including fish (Charlesworth and Mank 2010), amphibia (Uno et al. 2008) and reptiles (Vicoso et al. 2013), and there is convincing evidence that these systems evolved independently, as their sex chromosomes carry different sets of genes (Kawai et al. 2007; O'Meally et al. 2010; Quinn et al. 2011). The same is true in insects; for instance, the XY systems in Diptera are nonhomologous (Toups and Hahn 2010; Pease and Hahn 2012; Vicoso and Bachtrog 2015). Other animal taxa have female heterogamety, with ZW systems, as in birds (Zhou et al. 2014), Lepidopteran insects (Suetsugu et al. 2013) and Crustacea (Juchault and Rigaud 1995; Volpi et al. 1995), and these too often evolved independently, although changes from XY to ZW are also known (Ogata et al. 2008), and systems with males ZZ and females with a single Z have been detected in Dipteran flies, which mostly have XY systems (Vicoso and Bachtrog 2015). In all these taxa, at least some species’ sex chromosomes have physically large nonrecombining regions that have characteristics differing from most of the rest of the genomes. The nonrecombining regions in the heterozygous sex (Y or W chromosomes) are sometimes heterochromatic, with many genes missing that are present on the homologous X or Z chromosomes. The difference in the number of gene copies between the sexes is routinely used to discover fully sex‐linked regions (Vicoso et al. 2013; Zhou et al. 2014; Vicoso and Bachtrog 2015).

The ancestral states for these sex chromosome systems are generally not known, but many animal sex‐determining systems probably evolved by changes in pre‐existing sex‐determining systems (takeovers), which are predicted to occur in several situations (Bull 1983; van Doorn and Kirkpatrick 2007; Vuilleumier et al. 2007; Blaser et al. 2013) and are known in several anima taxa, including insects (Wilkins 1995; Beye et al. 2003) and fish (Ross et al. 2009; Schultheis et al. 2009; Myosho et al. 2012; Vicoso and Bachtrog 2015). Sometimes the ancestral state may be environmental sex‐determination, which is common in fish (Charlesworth and Mank 2010) and reptiles (Gamble et al. 2015).

Sex chromosomes have clearly also evolved repeatedly among flowering plants, although species with separate sexes (dioecy) are infrequent (Westergaard 1958; Charlesworth 1985; Renner 2014). Several plants with genetic sex‐determination have XY sex chromosome systems, and a few are ZW, and genetic studies indicate male or female heterogamety in some plants without cytologically detectable sex chromosomes (Westergaard 1958). Use of genetic markers has now revealed fully sex‐linked genes in several plant species. The fully sex‐linked region of *Silene latifolia* includes hundreds of genes, many still present on the Y chromosome (Bergero and Charlesworth 2011; Chibalina and Filatov 2011). Other species have few fully sex‐linked genes, for example the grape vine (Picq et al. 2014) and persimmon (Akagi et al. 2014), while some are intermediate between these extremes; for example, between 50 and 100 genes are sex‐linked in papaya, and about 50 have copies on both the X and the Y (Wang et al. 2012).

These differences in the sizes of the nonrecombining regions, and the numbers of genes included in them, may roughly correspond with differences in the ages of these sex chromosome systems. The ages can be estimated using DNA sequence divergence; when recombination stops, the Y‐linked alleles start accumulating substitutions in their sequences (see Box 2 figure), becoming increasingly diverged from their X‐linked alleles, and a molecular clock can be applied to estimate the time in years. The *S. latifolia* Y chromosome is estimated to have stopped recombining about 5–10 MYA (Nicolas et al. 2005), and the papaya Y‐linked region is slightly younger (Wang et al. 2012). Plants with highly heterochromatic Y chromosomes, including *Rumex acetosa* (sorrel) in the Polygonaceae (Shibata et al. 2000; Mariotti et al. 2008) and *Humulus lupulus* (hops) in the Cannabaceae (Westergaard 1958) may have older, systems, but they have not yet been firmly dated in this way. In one strawberry species, *Fragaria virginiana*, two closely linked sex‐determining genes have been found (Spigler et al. 2008), but a closely related species has suppressed recombination (Goldberg et al. 2010).

Consistent with the young ages just mentioned, dioecious plants often have close nondioecious relatives (Westergaard 1958; Charlesworth 1985; Renner 2014), indicating recent evolution of their sex chromosomes compared with those of mammals or birds, or some flies some of which have probably been established for over 200 million years (Vicoso and Bachtrog 2015).

The ancestral states of such young plant sex chromosomes can therefore often be inferred, and it is clear that flowering plants have evolved dioecy from both hermaphroditic ancestors (whose flowers have both male and female sex structures, the stamens and pistils, respectively) and monoecious ones, with individual flowers being unisexual, but both male and female flowers carried by each plant (Darwin 1877; Renner 2014). I will use the term ‘cosexual’ for these nondioecious systems, as they are nonunisexual at the individual plant level. The corresponding term in animals is hermaphroditic, as monoecious species do not occur, and dioecy is often called gonochorism (Maynard Smith 1978).

The combination of known ancestral states, and recent evolution, makes plants excellent for studying early stages of sex chromosome evolution, including recombination suppression. As in animals, some plant sex chromosomes have evolved nonrecombining regions that successively expanded, forming ‘evolutionary strata’ of different levels of sequence divergence between Y‐ and X‐linked alleles (Lahn and Page 1999). Regions of sex chromosomes that still pair and recombine are called pseudo‐autosomal regions (PAR), and these are the least diverged, while regions far from the PAR in the genetic map of the X chromosome form the older strata (Lahn and Page 1999; Skaletsky et al. 2003). Evolutionary strata are also found in bird sex chromosomes (Wright et al. 2014; Zhou et al. 2014), and two strata have been detected in both plant species whose X–Y sequence divergence has so far been studied, *S. latifolia* (Bergero et al. 2007) and papaya, *Carica papaya* (Wang et al. 2012).

**Figure 2:**
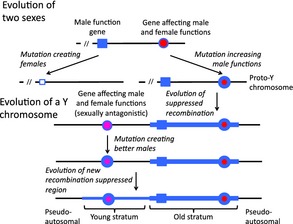
The evolution of separate sexes from an initial hermaphrodite. As in the case of a mimetic morph, two sexes could evolve by two or more changes. The first mutation creates females (symbolized by a change from a large filled blue square to a small open square), and subsequent mutations at two loci (circles) increase the male function of the nonfemale individuals (those with the large filled blue square allele), while decreasing their female functions (symbolized by increased amounts of blue and smaller amounts of red colouring of the alleles present at these loci). These mutations can be regarded either as female suppressors or as modifiers of the balance between male and female functions. There is thus similarity with the model for the evolution of Batesian mimicry in Fig. 1A, with modifiers that are expressed regardless of the individuals’ gender (rather than specifically in individuals with one morph/sex, as in Fig. 1B). The chief difference from the Batesian mimicry case is that, in the evolution of mimicry, both mutations occur on the same ‘mimicry haplotype’, whereas, in the case of separate sexes, the male‐sterility mutation (creating females) occurs in a male function gene carried on one member of a homologous chromosome pair, while the male‐promoting/female‐suppressing mutations must occur on the other homolog, otherwise a sterile phenotype would be produced, causing the selective loss of the second mutation.

The evolution of dioecy in plants exemplifies the supergene hypothesis for sex chromosomes, as it clearly involves situations like the general case shown in Box 2 that must promote the evolution of suppressed recombination. During the evolution of dioecy from a cosexual state, two or more mutations must invade the population (Fig. 2), first when dioecy initially evolved, and later as males and females evolved in the absence of constraints imposed by the other sex functions. My own work has largely concerned the initial stages of the evolution of separate sexes, but recently has also considered the later stages, which are of particular interest, because the processes involved probably also occur in situations where dioecy evolved long ago, and after takeover events that created new single gene sex‐determining systems as in many animals.

The right‐hand side of Fig. 2 shows that the initial change from cosexuality to separate sexes in a plant requires at least two mutations, one creating females (the change at the top left in Fig. 2), and then one or more female‐suppressing mutations on the homologous chromosome, creating males or male‐biased cosexuals (Westergaard 1958; Charlesworth and Charlesworth 1978). It is the development of LD between these mutations (with different allele frequencies in the two sexes, Charlesworth and Charlesworth 1980) that generates the selection for recombination suppression. This LD is generated and maintained by selection against recombinants with both a male‐sterility factor and a male enhancer that reduces female functions.

Despite this disadvantageous situation that results during the evolutionary change from cosexuality to dioecy, each of the mutations spreads, due to an advantage. The first mutation (a recessive or largely recessive male‐sterility mutation creating females) can spread, despite the loss of male functions, if it increases overall fitness through (i) increasing the frequency of offspring produced by cross‐fertilization, which will not suffer from inbreeding depression and (ii) making more resources available for seed production (Lloyd 1975; Charlesworth and Charlesworth 1978). As females cannot become fixed in a population, the mutation will become polymorphic. A second mutation may then arise that changes the initial cosexes into males (i.e. a female‐suppressing mutation). As in the case of mimicry, this might affect only the cosexual, pollen‐bearing portion of the population (which it benefits, allowing the mutation to go to fixation). Sometimes, however, females may also be affected, and their female functions will then be abolished. Under these assumptions, a nonsex‐limited mutation creating males is sexually antagonistic, as illustrated in Fig. 2. Such male‐enhancing mutations therefore have the properties of the female suppressors that have been detected in genetic experiments with dioecious plants (Westergaard 1958) and in Y chromosome deletion experiments in *S. latifolia* (Lardon et al. 1999; Zluvova et al. 2005; Bergero et al. 2008; Fujita et al. 2011). Mutations that make the initial cosexual phenotype more male‐like (rather than fully male) may also often involve sexually antagonistic ‘trade‐offs’, particularly if the ancestral state is monoecy, because increasing the investment in flowers of one sex probably decreases that invested in the other sex.

In theoretical models, such male‐enhancing/female‐suppressing mutations can spread in populations polymorphic for females and the initial cosexual phenotype, unless they are highly recessive. Increased maleness is more advantageous when females are present than when they are not, although a larger increase in maleness is needed than the decrease in the seed production of the females (Charlesworth and Charlesworth 1978). As it is clearly very disadvantageous to have both a male‐sterility mutation and a nonsex‐limited female suppressor/male enhancer mutation, the latter mutation is able to spread only if it is sufficiently closely linked to the locus that mutated to the male‐sterility allele (just as in the supergene model for mimicry, close linkage minimizes the conflict because most individuals have the successful phenotypes). Polymorphisms for both loci can then arise, with closer linkage favoured (Bull 1983).

The spread of a male‐enhancing/female‐suppressing mutation may be followed by more sexually antagonistic (SA) mutations. During the evolution of dioecy from cosexuality in plants, full maleness often evolves in several steps (see Fig. 2); this may be similar to evolution from a crude mimetic resemblance to perfect mimicry, involve several steps. Many plants are ‘subdioecious’ – their pollen‐bearing individuals have some female function (for example, producing fruits in favourable conditions), suggesting that they have not yet completely suppressed female functions. Gradual evolution of full maleness may help explain sex chromosomes’ observed evolutionary strata.

Even when dioecy has evolved (and males no longer have any female functions), mutations improving male function may still occur and may again have side effects reducing female functions. Such situations could also arise during the evolution of animal sex chromosomes (Beukeboom and Perrin 2014). Theoretical models of mutations benefitting one sex at the expense of the other show that they can establish polymorphic states (rather than spreading throughout the population) most readily at loci closely linked to the sex‐linked region (Rice 1987; Jordan and Charlesworth 2012), again creating selection for reduced recombination with the sex‐determining region.

The trade‐offs and conflicts assumed in these models are hypothetical, and more work is needed to test whether they were actually involved in the evolution of sex linkage. The best current evidence comes from a fish, the guppy (*Poecilia reticulata*), whose males are polymorphic for characters that are advantageous during courtship (colour, number, shape, size and position of spots), but that make them conspicuous to predators (Gordon et al. 2012). This fish has genetic sex‐determination, and many genes controlling presence/absence of colour pattern elements are fully Y‐linked (and therefore restricted to males). Some coloration genes, however, are partially sex‐linked and not phenotypically expressed in wild females (Haskins et al. 1961; Lindholm and Breden 2002). These findings strongly suggest that the male characters are sexually antagonistic, with the partially sex‐linked alleles either being male‐specifically expressed mutations or having evolved male‐specific expression to avoid harm to females. Trade‐offs between mating advantages to males and higher predation risk can potentially maintain polymorphisms for coloration alleles, and indeed frequencies of coloration are low in populations with high predation rates (Fisher 1930). Recombinant females may also be commoner in populations with low predation rates. When females are changed into males by tesosterone treatment, to reveal those carrying non‐Y‐linked coloration alleles (probably mostly partially sex‐linked), females carrying coloration genes were consistently commonest in populations with low predation rates (Gordon et al. 2012). This could be due to a more frequent recombination in such populations, although this remains to be tested.

Sexual selection is only one way that sexually antagonistic selection could arise. Sex differences in physiological requirements are less easy to study because visible characters are not involved, but quantitative trait locus (QTL) analysis can potentially detect such variation. This approach detected several QTLs in the PAR region of the plant *S. latifolia*, as well as autosomal QTLs. Like the partially sex‐linked genes in guppies, the PAR QTLs appeared only in the males, consistent with a past conflict between the sexes that has been resolved by evolution of sex‐limited expression of the characters. There is also some evidence for polymorphisms in this plant's PAR region that could select for suppressed recombination (Qiu et al. 2013). The main caveat to the conclusion that sex chromosomes are probably supergenes is the current lack of direct evidence that recombination suppression is selectively driven and that sexually antagonistic selection is involved. Despite there being no obvious plausible alternative to selection having led to suppressed recombination, alternatives may exist, and the sexually antagonistic polymorphism hypothesis should therefore be further tested.

## Flowering plant self‐incompatibility

Another situation where the evolution of supergenes has been proposed is plant self‐incompatibility (SI). In self‐incompatible plants, self‐fertilization is prevented by inhibiting growth of pollen tubes of the same ‘incompatibility type’ (including self‐pollen) before fertilization. Supergenes might be expected to be involved in the genetic control of SI because separate genes may control the stigma and pollen incompatibility reactions.

### Homomorphic self‐incompatibility: systems with no evolved supergene

Although systems with a single gene may be possible in homomorphic SI systems (where the flowers of the different incompatibility types are morphologically indistinguishable), it molecular studies in plants such as Brassicas, Nicotianas and poppies have now demonstrated that one gene controls the stigma incompatibility type, and one or more separate genes control the type of the pollen (Schopfer et al. 1999; Takayama et al. 2000; Charlesworth et al. 2005; Wheeler et al. 2009; Kubo et al. 2010). For example, in several *Brassicaceae* species, including *Arabidopis lyrata*, the initial step of the self‐pollen‐rejection pathway involves interaction between a receptor kinase expressed in stigmas of the flowers, and a ligand on the pollen surface. Incompatibility requires the correct allele combinations of the pollen and stigma genes, that is LD between them must be maintained – genotypes with recombinant allelic combinations would be self‐compatible, leading to loss in fitness due to inbreeding depression.

In *Brassica* species, and *A. lyrata*, as well as in self‐incompatible species in the distantly related family, Rosaceae, the SI genes are physically close to each other (Kusaba et al. 2001; Entani et al. 2003). However, this does not imply that recombination rates have evolved to be low. In the *A. lyrata* SI region, recombination occurs immediately outside a physically small region that includes only the two genes controlling the pollen and stigma incompatibility types (Goubet et al. 2012). Therefore, the incompatibility genes could have evolved from two loci that were already adjacent before they evolved their roles in SI, as in case A in the figure in Box 2. This is supported by the recent discovery that SI in the genus *Leavenworthia* also involves two closely linked genes, which resemble those in *Brassica* and *A. lyrata*, but are paralogs, not orthologs, and evolved on a nonhomologus chromosome (Chantha et al. 2013). The *Leavenworthia* chromosomal homolog carrying the incompatibility genes of *Brassica* and *Arabidopsis* species also carries homologs of this pair of loci. These are not linked to the *Leavenworthia* incompatibility locus, and their allele sequences diverged after the *Leavenworthia* lineage split from the other Brassicaceae. These results suggest that different lineages evolved incompatibility independently, from already linked loci, with no supergene evolution (Chantha et al. 2013).

### Distylous plants

However, supergenes may well have evolved in distylous species, such as many *Primula* species. Distyly is a form of heteromorphic SI in which the flowers of the two or three different types differ morphologically, with the stigmas in each morph being distant from the anthers, but at similar positions to the anthers in another morph's flowers (Darwin 1877). Plants with two morphs are called ‘distylous’, and species with three different flower morphs are called ‘tristylous’ (reviewed by Barrett 2013).

The long‐styled versus short‐styled differences of distylous plants are controlled by a single genetic locus (reviewed in Barrett 1992). In *Primula*, different flower developmental characters are involved, and they seem unlikely to be controlled by a single gene: as well as the length of the style, and the placement of the anthers, these include the stigma surface morphology and the pollen coat, and the incompatibility types. It has therefore been suggested that a supergene of at least three distinct genes controls distyly. Short‐styled plants have been proposed to have the genotype *S*/*s*, where *S* represents a ‘haplotype’ *S *= *GPA* carrying dominant alleles *A* (for the high anther position), *P* (for the incompatibility type of that anther position) and *G* (for the short‐styled state and incompatibility type), while long‐styled plants are *s*/*s*, where the *s* (= *gpa*) haplotype carries recessive alleles for the opposite respective characters.

In support of the supergene hypothesis, ‘homostyle’ plants, whose flower phenotypes combine features of the two different morphs, are occasionally found and are inherited as alleles of the same locus (Haldane 1933), suggesting that control of different flower characters involves distinct genes occasionally separable by recombination. Homostyles include such genotypes as gPA (homozygotes or *gPA*/*gpa* heterozygotes, or *S*_h_/*s*), which have high‐level anthers and the pollen incompatibility type appropriate for high‐level anthers, but also high‐level stigmas and the appropriate stylar incompatibility type for the short‐styled morph, such that these plants are self‐compatible (Crosby 1949; Piper et al. 1984).

The ancestral states, and the selective advantages involved in the changes that occur during the evolution of distyly, are much less clear than for mimicry and sex chromosomes. Ancestral character states can be reconstructed using estimated phylogenetic trees, but this is complicated by incomplete sampling of extant taxa, extinctions and uncertainty about to how to weight gains versus losses of distyly (see Barrett 2013). The first attempt to model the changes assumed that the ancestral flower had long styles and anthers at a matching position (called ‘long homostyle’). The evolution of distyly was assumed to involve initial evolution of a SI system with two incompatibility types, followed by a changed anther position to avoid wastage of pollen by its transfer to incompatible stigmas, including stigmas of the same flower. Once an anther position variant had become established, a stigma position (style length) variant would benefit from improved pollen receipt from anthers in the new (variant) position, potentially optimizing reciprocal pollination of each morph by the other and leading to distyly. Just as in the cases already discussed, the variants in this scenario each gain an advantage only in combination with the appropriate variants in other characters, so that the system evolves only when mutations appear in linked genes, and selection will favour closer linkage (Charlesworth and Charlesworth 1979a).

The true ancestral state, and therefore the selective advantages of the changes involved, may well differ from this scenario (Lloyd and Webb 1992a,b), and indeed distyly might evolve from various ancestral states. The ancestor may have been long‐styled, as is common in flowering plants (Lloyd and Webb 1992a,b), and as inferred by the only character state reconstruction so far, a set of disylous species in the genus *Exochaenium* (Gentianaceae) (Kissling and Barrett 2013). Some of the variants involved may also have affected multiple characters, such as both style length and incompatibility type (Charlesworth and Charlesworth 1979a,b; Lloyd and Webb 1992a,b), rather than distinct genes for each. Nevertheless, interactions are likely, and the spread of each of the mutations involved probably depended on those that had already become established, so that suppressed recombination would have been favoured.

Given these uncertainties it is particularly interesting to renew studies of the genetics of distyly, using modern molecular markers, which can give high enough marker densities to test whether a nonrecombining region is present at the distyly locus, or not, and how large a genome region is involved. Approaches that have been used to study sex chromosomes should be valuable for studying distyly, including identifying variants specific to the dominant S haplotype (controlling short style length, which is always heterozygous in distylous populations), just as male‐specific variants indicate Y‐linkage. Sequence divergence between the two haplotypes will then show how long they have been nonrecombining (like the strata in sex chromosomes). Efforts have therefore been made to identify genes linked to the S‐locus in distylous plants. In *Turnera* (Labonne et al. 2008, 2009; Labonne and Shore 2010), complete linkage has not been firmly established for any gene.

Two genes completely linked to the distyly locus have, however, been identified in buckwheat (*Fagopyrum esculentum*), and studies of plants from natural populations showed that some variants in these genes are specific to the *S* allele, which is strong evidence that recombination with the long style allele (*s*) is very rare (Yasui et al. 2012). This study sequenced about 610 kb around one of the genes, which included at least one other gene and also repetitive transposable element sequences; the repeats make assembly difficult, and the fully S‐linked region must be larger than the total length of current contigs. This region could therefore include genes other than those controlling distyly and might resemble a sex‐linked region of moderate extent such as that of papaya (see the figure in Box 2).

Recent results including assembled genome sequence data in *Primula* are also pointing to the conclusion that an extensive region may be fully linked to the heterostyly locus (Nowak et al. 2015). Bulk segregant analysis in a *Primula veris* (cowslip) family found sequences containing variants specific to the short‐styled morph. Although this approach will probably not ascertain all linked loci, as inferences must be very conservative to avoid including too many false‐positive results, 13 variants (single nucleotide polymorphisms (SNPs)) in candidate S‐linked linked loci/sequences were validated. In addition, the reliance on genome assembly may miss potentially diverged *S* haplotype sequences, especially if the *S* and *s* haplotypes are rearranged with respect to one another like X versus Y chromosomes, or the haplotypes seen in Batesian mimicry loci (see above). Nevertheless, this is an important advance, because finding some candidates makes it possible to test whether they are located in a physically small genome region, or spread across a physically large region. To find genes for such tests (and to help identify the flower development genes involved), transcriptomes of both morphs were sequenced, and genes with differing expression in the two morphs were studied further (although the genes controlling heterostyly might not differ in expression, so this may have narrowed down the pool of candidates). Highly diverged sequences could either be paralogs or S‐linked alleles. Genetic tests validated six genes as fully or partially linked to the S‐locus, using tests for variants specific to the short morph in a sample from a natural population (Nowak et al. 2015).

Distyly is old‐established in the genus *Primula* (Mast et al. 2006), so, if a nonrecombining region exists, the two haplotypes may have been isolated for a long evolutionary time. Like males in an XY sex chromosome system, *Ss* (short‐styled) plants should be heterozygotes for the entire *S *=* GPA* and *s *= *gpa* haplotypes described above, and genes other than those controlling the flower phenotypes should also show high between haplotype sequence divergence (as between old‐established X and Y chromosomes). Therefore, a sample of alleles of any gene from the region should show high nucleotide diversity (per site expected heterozygosity), because alleles from both haplotypes will be included (see case C in the figure in Box 2); other genome regions will not have old ancestry and will have lower diversity. Because they are heterozygotes for two diverged haplotypes, short‐styled individuals should also show higher diversity in the S‐locus region than long‐styled ones, which are homozygous for the *s* haplotype.

To estimate diversity, as an indication of *S*‐*s* interhaplotype divergence, the *Primula* study computed S‐morph/L‐morph SNP ratios per scaffold. S‐locus‐linked scaffolds gave ratios not much higher than the genomewide values (Nowak et al. 2015). This is unexpected: even if the chromosome carrying the S‐locus has exceedingly high diversity (due to an extremely long *S*‐*s* divergence time), this should not be detected in the genome as a whole unless this chromosome region is very large. Moreover, extreme divergence will make it very hard to ascertain both alleles of such a region (see above), so such genes are unlikely to have been included in this analysis.

Nevertheless, the success in finding S‐linked genes suggests that the region was easy to find, which in turn suggests that it may be physically large, and that the haplotypes’ sequences are probably highly diverged. At present, it is also unclear whether sequence variants associated with the S and s haplotypes are spread across a physically large nonrecombining region. In the study, four fully S‐linked markers were found in three different scaffolds (of 106, 186 and 327 kb), consistent with suppressed recombination affecting an S‐locus region big enough to include multiple genes other than those controlling the distyly. In the future, it will be interesting to test whether or not recombination suppression is similar in all *Primula* species, and whether homostyles and homostylous species (Mast et al. 2006) show evidence of being rare recombinants.

## Other linked systems

Consistent with the supergene hypothesis, the three examples discussed in detail here have the common feature that at least two different genes seem likely to have been involved, as the phenotypes seem unlikely to evolve by single genetic changes. Instead they seem likely to be built up by a succession of mutations that are not unconditionally advantageous, occurring in interacting genes spreading in an initial population, such that trade‐offs lead to build‐up of adapted polymorphic gene complexes that generate selection for suppressed recombination. However, it is worth outlining some examples where further work may show that suppressed recombination has evolved for similar reasons and also a situation where suppressed recombination clearly evolved in a different context.

Perhaps the best understood case that closely resembles those outlined above is segregation distortion, where alternative haplotypes carry distorter alleles along with alleles conferring protection from the distorter, or nondistorter alleles susceptible to distortion (Charlesworth and Hartl 1978). Recombination is clearly disadvantageous in such situations. Several cases in different organisms have indeed been found to involve the evolution of genome regions with suppressed recombination (Lyon 2003; Dyer et al. 2007).

Another case in which suppressed recombination allows LD between polymorphic loci is the chromosome controlling divergent forms of social organization in fire ants (Wang et al., 2013). The two forms, monogyne or polygyne, also differ in many other biologically important respects, suggesting evolution of a complex adaption. At least one inversion, of around 9 megabases, contributes to suppressed recombination in heterozygotes across more than half of the chromosome pair, and the two haplotypes in this region are associated with inheritance of most phenotypic differences between individuals of the two social forms.

Yet another intriguing case is the complex inversion system in the white‐throated sparrow. The two haplotypes control different traits, which include plumage differences between two morphs (in both sexes), preferential mating with the other plumage morph, and aggressiveness (Huynh et al. 2011). Again a succession of adaptive changes may therefore have evolved, but there is no evidence for trade‐offs nor is it known why linkage might have been favoured in this case.

Speciation may sometimes involve situations that select for suppressed recombination in order to maintain LD between alleles adapted to two different environments (Faria and Navarro 2010). This can also occur in clines (Charlesworth and Charlesworth 1979b). In self‐fertilizing species, such as *Caenorhabditis elegans*, adaptation could involve coadaptation with other genes in the genome, leading to incompatibility with other lineages of the same species, and genetic evidence supports the hypothesis that the incompatibilities are a consequence of complex interactions between multiple loci (Snoek et al. 2014). In one case, two interacting genes involved have been mapped to a small genome region (Seidel et al. 2008). This is an area of active current research.

Some fungal mating‐type systems involve interactions between genes encoding pheromones and their receptors (Casselton 1998), similar to the ligand‐receptor systems in flowering plant homomorphic SI outlined above. However, there is no evidence that close linkage has evolved in these systems to maintain correct combinations of these components.

In contrast, some highly self‐fertilizing species of fungi have nonrecombining the mating‐type regions. These evolved under selection without interactions such as those discussed above. Close linkage of the mating‐type locus to the centromere guarantees segregation in the first meiotic division, so that, after meiosis, each sexual spore has nuclei of opposite mating types, and can mate (whereas if a crossover occurs in the region, the progeny would not be self‐fertile). Such ‘pseudo‐homothallic’ systems are known in *Neurospora tetrasperma* (Jacobson 2005; Ellison et al. 2011) and *Microbotryum lychnidis‐dioicae* (Garber and Ruddat 2002; Hood and Antonovics 2004). These cases resemble sex chromosome evolution only in the sense that genes involved in mating cause selection for suppressed recombination, but the reasons are very different. The challenge in studying mating‐type regions in such fungi is to determine whether inversions caused suppressed crossing over, or whether recombination suppression evolved first, allowing inversions to accumulate in one haplotype or the other (as, if recombination does not occur, inversions no longer lead to the risk of chromosome breaks). In mammalian sex chromosomes, it is clear that, although inversions may have caused initial recombination suppression (Lahn and Page 1999; Lemaitre et al. 2009), inversions also accumulated after suppressed recombination evolved (Hughes et al. 2010).

Studying different systems is likely to continue to provide greater understanding of gene clusters in the future, and, as already mentioned, molecular evolutionary approaches used to study sex chromosomes should become valuable for studying the other systems. The main surprise from the new work Batesian mimicry (and perhaps distyly) is that supergenes, as originally conceived, may not have evolved, and there are certain to be other surprises in the future.
